# Lectures on Diseases of Women

**Published:** 1881-09

**Authors:** 


### BOOK NOTICES AND REVIEWS.

Lectures, Clinical and Didactic, on the Diseases of Women. By *R. Ludlam, M. D.*, Professor of the Medical and Surgical Diseases of Women, in the Hahnemann Medical College and Hospital, of Chicago, etc., etc. Fifth Edition, revised, enlarged and illustrated. pp. 1028; price, cloth, $6.00. Chicago: Duncan Brothers. 1881.

Gynæcology, in the Old School, may be divided into two branches; the surgical and the therapeutic or medical. From an allopathic point of view, both of these classes are wrong--the one relying too much on surgical measures, the other too entirely on medicinal. From a homœopathic standpoint, it is evident that surgical measures should be entirely subordinated to the therapeutic, and should be used only as a last resort. Under the lead of Simpson, Wells, and others, in England; of Simon, Esmarch, etc., in Germany; of Atlee, Sims, etc., in America, gynæcology has become a surgical art. There is an excessive surgical tendency exhibited by the leading gynæcologists of the Old School. As allopathy has no therapeutics, and, as Dr. Matthews Duncan says, "tangible remedies are the favorites of the physician and the vulgar," one is not surprised at their relying so much on surgical measures. But, among homœopathists we should expect to see pre-eminence given to internal medication. We are, therefore, pained to observe in this large work of Prof. Ludlam, how entirely he ignores the internal medication, which has achieved such success in the hands of our leading practitioners, for the surgical measures of allopathy. Although mention is made of internal medication and remedies given, this part of his treatment is evidently considered as of minor importance. As an instance, on page 307, in discussing the subject of "nausea and vomiting of pregnancy," we read: "For the vomiting of a viscid mucus, especially on rising, *Nux vomica* and *Cocculus*. For constant, or occasional vomiting, without regard to the position of the body, and for vomiting of whatever is swallowed, egesta being mixed with bile or mucus, *Ipecacuanha*. * * * For the vomiting of bile with the food, a rancid heart-burn, and ptyalism, especially at night, *Mercurius*." After giving a small list of other remedies, "special indications, for which you will look to the materia medica," our author adds: "The number and variety of these remedies implies that the so-called morning sickness of pregnancy is a self-limited disorder, because [!] when a disease inclines to get well of itself, it may easily happen that whatever has been prescribed will sometime or other get the credit of having cured it." This sentence betrays the author's lack of faith in homœopathy and its medicines, and also his ignorance of homœopathy. He had as well say that *consumption* is a self-limited disease, because many remedies are used in its treatment. It betrays ignorance of homœopathy, because the number of remedies, whose symptoms apply to any disease, have nothing whatever to do with its curability or non-curability.

Dr. Ludlam's book includes a wide range of subjects: the functional, diseases of menstruation, of pregnancy, hysteria, etc., as well as the organic diseases of the ovaries, uterus, etc. The work is well and profusely illustrated; its descriptions are brief and good, but it displays rather the character of a compilation than the stamp of originality, such as observed in the treatises of Thomas, Emmet or Barnes. By the way, in several instances, the author's quotations are taken from older editions of the writers quoted. It would have been better to have given their latest and ripest experience. In conclusion, we again express our regret that Dr. Ludlam has so entirely ignored homœopathy and its remedies for the useless expedients of allopathy. His work can in no sense be called homœopathic, nor is any such claim made. Hahnemann and homœopathy are conspicuous only by their absence. As an allopathic work, it cannot rank with those of Thomas or Emmet; as a homœopathic work, it is immensely inferior to Jahr or Guernsey.

